# Notch Signaling Function in the Angiocrine Regulation of Tumor Development

**DOI:** 10.3390/cells9112467

**Published:** 2020-11-12

**Authors:** Alexandre Trindade, António Duarte

**Affiliations:** CIISA-Centro de Investigação Interdisciplinar em Sanidade Animal, Faculdade de Medicina Veterinária, Universidade de Lisboa, Avenida da Universidade Técnica, 1300-477 Lisboa, Portugal; aduarte@fmv.ulisboa.pt

**Keywords:** Notch, Delta-like, Jagged, angiogenesis, tumor, angiocrine

## Abstract

The concept of tumor growth being angiogenesis dependent had its origin in the observations of Judah Folkman in 1969 of a retinoblastoma in a child. Tumor angiogenesis is initiated when endothelial cells (ECs) respond to local stimuli and migrate towards the growing mass, which results in the formation of tubular structures surrounded by perivascular support cells that transport blood to the inner tumor. In turn, the neo-vasculature supports tumor development and eventual metastasis. This process is highly regulated by several signaling pathways. Central to this process is the Notch signaling pathway. Beyond the role of Notch signaling in tumor angiogenesis, a major hallmark of cancer development, it has also been implicated in the regulation of tumor cell proliferation and survival, in epithelial-to-mesenchymal transition, invasion and metastasis and in the regulation of cancer stem cells, in a variety of hematologic and solid malignancies. There is increasing evidence for the tumor vasculature being important in roles other than those linked to blood perfusion. Namely, endothelial cells act on and influence neighboring tumor cells by use of angiocrine factors to generate a unique cellular microenvironment, thereby regulating tumor stem-like cells’ homeostasis, modulating tumor progression, invasiveness, trafficking and metastasis. This review will focus on Notch signaling components that play a part in angiocrine signaling in a tumor setting.

## 1. Introduction: Angiogenesis

The first description of the process of angiogenesis was made in 1794 by a British surgeon and anatomist, John Hunter [1]. Angiogenesis refers to the formation of new blood vessels from pre-existing ones [2] and requires a series of complex remodeling processes such as: vasodilation, cellular permeability, peri-endothelial support, proliferation, migration, lumenization, survival, differentiation and remodeling [3]. Triggering angiogenesis requires a disruption between the fine balance of pro-angiogenic and anti-angiogenic molecules [4]. It is initiated through endothelial basal membrane and extracellular matrix degradation of the pre-existing vessels, which is mediated by metalloproteases [5]. A new matrix is then synthesized by stromal cells and laid down, which together with soluble growth factors, enables the migration and proliferation of endothelial cells [6]. The vascular endothelial growth factor A (Vegfa) determines the migration direction, which is performed by specialized endothelial cells named tip-cells [7]. After sufficient endothelial cell division and migration has occurred, endothelial cells arrest in a monolayer and form a tube-like structure. Mural cells (pericytes and smooth muscle cells) are then recruited [6] for maturation of the newly formed vessel. Finally, if proper angiogenesis took place, blood flow is then established in the new vasculature. The angiogenic process occurs during embryonic development [3], and, in the adult, it can occur in physiological situations, like the oestrus cycle in the females [8], or in wound healing situations [9], whereas in adult pathological scenarios it occurs in tumor growth [10], among others [11].

The experimental study of new blood vessels formation, i.e., angiogenesis, began in the late 1930s and early 1940s, when several investigators studied the events of neovascularization in experimental tumors [12,13]. In these experiments, tumors were separated from host tissue by a micropore filter and demonstrated that an unknown diffusible substance was released from the tumor and could stimulate new blood vessel growth. However, prior to 1970, the prevailing belief was that tumor angiogenesis was a side-effect of dying tumor cells. It was only in 1969 that Judah Folkman raised the possibility of tumor growth being angiogenesis-dependent by observing a retinoblastoma in a child, which consisted of a large tumor that protruded from the retina into the vitreous and was highly vascularized. Moreover, he observed tiny metastases shed in the vitreous that were all avascular and had a necrotic center. Therefore, he developed the concept that tumors could not grow beyond approximately 1–2 mm without recruiting new blood vessels. These findings were published in 1971 [10] and since then a rapidly increasing body of work in the field of angiogenesis allowed for the investigation and discovery of several molecular mechanisms involved in that process.

Since Folkman’s seminal discovery, many signaling pathways have been identified as key contributors to the neo-angiogenic process, leading to the creation and application of anti- angiogenic drugs in cancer treatment, such as the anti-VEGF antibody bevacizumab and the tyrosine kinase inhibitors like sunitinib or sorafenib, among others [14,15,16].

## 2. Angiocrine Signaling

**Figure 1:**
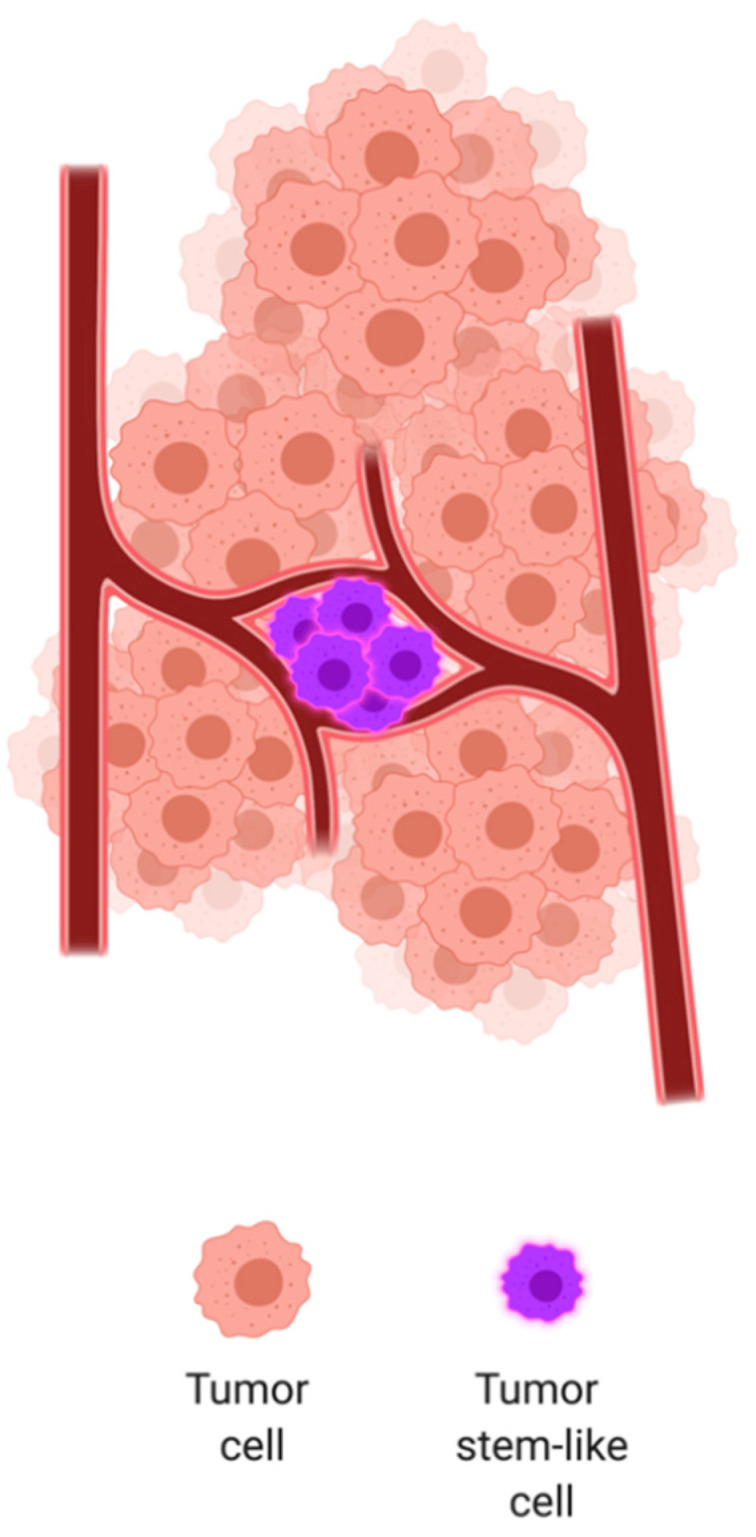
The tumoral stem cell perivascular niche. Tumor stem-like cells are commonly found in the perivascular space. Although we still do not fully know the reasons why, the causes are usually attributed to metabolic needs and dependency on molecular signals coming from tumor endothelial cells.

Endothelial cells do not only serve as building blocks for new vessels, but they can also provide signaling molecules and secreted growth factors that maintain organ homeostasis, balance the self-renewal and differentiation of stem cells and orchestrate organ regeneration and tumor growth in a perfusion-independent manner. This novel type of instructive, vascular-controlled cell–cell communication was proposed by Shahin Rafii in 2010 and referred to as angiocrine signaling [17,18]. Angiocrine factors comprise secreted and membrane-bound inhibitory and stimulatory growth factors, trophogens, chemokines, cytokines, extracellular matrix components, exosomes and other cellular products that are supplied by tissue-specific endothelial cells to help regulate homeostatic and regenerative processes in a paracrine or juxtacrine manner [19]. As angiocrine factors modulate the proliferation of stem and progenitor cells, it is conceivable that the vascular niche (Figure 1) might also directly modulate the homeostasis of tumor initiating cells [20].

## 3. Notch Signaling Pathway in Angiogenesis and Tumoral Neoangiogenesis

The neo-angiogenic process is regulated by many signaling pathways. Among them is the Notch signaling pathway, an evolutionarily conserved signaling system that regulates proliferation, differentiation, cell-fate determination, progenitor and stem-cell self-renewal, in both embryonic and adult tissues [21,22]. The Notch signaling pathway was first discovered about 100 years ago when John Dexter, in 1904, described Drosophila melanogaster variants displaying wing phenotypes now associated with Notch pathway mutations [23]. Three years later, Thomas Morgan was able to identify the mutant alleles [24], but it was only after the molecular biology revolution, that Spyros Artavanis-Tsakonas and Michael Young were able to clone the Notch receptor and thus attribute the wing-notching phenotype to gene haplo-insufficiency [25,26]. These initial studies created the basic foundation for a new era in various fields, including developmental and stem cell biology, neuroscience, and cancer biology [27]. Since then, the Notch signaling pathway has been extensively characterized in its role in cell-fate determination, differentiation, proliferation, progenitor and stem-cell self-renewal, in a diversity of embryonic and adult tissues [22,28].

The Notch pathway is composed of five ligands (Jagged1, Jagged2, and Delta-like 1, 3, and 4) and four receptors (Notch 1–4). Notch protein receptors reside on the cell surface as non-covalently linked heterodimers that are comprised of the extracellular and transmembrane (intracellular) Notch polypeptides. Extracellular portions are characterized by numerous epidermal growth factor (EGF)-like repeats. Transmembrane portions include the membrane-proximal RBP-J-associated molecule (RAM) domain, which mediates interaction with several cytosolic and nuclear proteins; the Ankyrin (ANK) domain, which is also important for protein–protein interactions; two nuclear-localization sequences (NLSs); a carboxy-terminal transactivation domain (TAD), which is important for activating transcription; and a PEST (proline-, glutamate-, serine- and threonine-rich) domain, which is important for regulating Notch degradation. Transmembrane Notch3 and Notch4 are shorter and lack the TAD. The heterodimerization domain (HD) spans the region of interaction between the extracellular and transmembrane portions. The Notch ligands contain an EGF-like repeat region and a conserved sequence also known as Delta/Serrate/Lag (DSL). Jagged1 and Jagged2 each have a conserved cysteine-rich (CR) domain [22].

The functional Notch receptors are translocated to the cell surface as processed heterodimers. The final heterodimeric form of the receptors is preceded by a series of transformations which include: a Furin-dependent cleavage (S1 cleavage) in the Notch extracellular domain (NECD), that occurs during trafficking through the Golgi complex [29]; and a glycosylation by O-fucosyltransferase and Fringe family N-acetylglucosaminidyl transferases that is crucial for proper folding of the Notch receptor and the interaction with ligand-specific DSL domains (Delta, Serrate, Lag-2) [30].

Distinct ligand affinities exist for the various receptors, altered by glycosylation, which influences downstream transcriptional activation [31]. Classical Notch pathway activation requires ligand-receptor in adjacent cells because the ligands remain immobilized as transmembrane proteins. After Notch receptor binding, the ligand undergoes endocytosis into the ligand-presenting cell, which causes a mechanical disruption of the Notch receptor by changing the conformation of the negative regulatory region of the receptor. This conformational change in the Notch receptors allows for a second cleavage (S2) of the ectodomain by an *Adam17* metalloprotease/*Tnf-α* converting enzyme (TACE) [32], followed by a third cleavage (S3) mediated by the presenilin-γ-secretase complex [33]. This sequence of cleavages leads to the release of the intracellular portion of the Notch receptor (NICD). The NICD contains nuclear localization signals (NLSs) within the RAM domain, which allows for the the translocation to the nucleus where it forms a complex with the inactive DNA-binding factor CSL/RBPjk (CBF1/Suppressor of Hairless/Lag1) and recruits other co-activator proteins from the Mastermind-like family of proteins such as MAML1 [34,35]. In the absence of NICD, RBP-Jk associates with a corepressor complex and acts as a transcriptional repressor of Notch target genes [36]. In turn, the NICD/RBP-Jk complex leads to the transcription of Notch downstream target genes, such as several helix–loop–helix transcription factors (*Hey* and *Hes* gene families among others) [22].

The study of Notch pathway components, specifically loss-of-function mouse mutants, has provided detailed information regarding the importance of these genes in the regulation of embryonic angiogenesis. Notch 1 is the most broadly studied Notch receptor and the main receptor responsible for Notch signaling associated phenotypes. Genetic deletion of *Notch1* in mice results in embryonic lethality by severe vascular and cardiovascular defects [37]. The *Notch2* gene was the second of the mammalian Notch family receptors to be cloned [38]. Later, mice homozygous for a hypomorphic *Notch2* mutation were reported to present defects in development of the kidney, heart and eye vasculature [39]. Notch2 was also shown to be expressed in vascular smooth muscle cells and to play a critical role in vascular maturation [40,41,42]. *Notch3* loss-of-function in mice resulted in profound structural and functional defects in arteries, due to impaired vascular maturation indicating a potential role in smooth muscle cell differentiation [43]. Notch4 is primarily expressed on the endothelium and the endocardium [44] and genetic deletion of *Notch4* exacerbated the embryonic lethal vascular defects associated with Notch1, even though it did not produce a detectable phenotype on its own [45,46], suggestive of an important role in vascular development. Dll1 was shown to be essential for post-natal arteriogenesis [47] and established as a critical endothelial Notch ligand required for maintaining arterial identity during mouse fetal development [48]. Jagged1 is expressed in endothelial and vascular smooth muscle cells [49]. *Jag1*-null mouse mutants die at 11.5 dpc because of heart defects and abnormal development of the yolk sac and head vasculature [50]. Dll4 is the most broadly studied Notch ligand in vascular biology. The Dll4 ligand was first described as a vascular endothelium specific ligand [51]. In the developing embryo, expression of Dll4 is initially restricted to large arteries, whereas in adult mice its expression is limited to small arteries and capillaries [52]. Haplo-insufficiency of *Dll4* in mice resulted in embryonic lethality at approximately 10.5 dpc due to defective vascular development, including abnormal stenosis and atresia of the aorta, defective arterial branching from the aorta, arterial regression, gross enlargement of the pericardial sac and failure to remodel the yolk sac vasculature. These studies revealed Dll4 to be essential for arterial patterning and vascular remodeling during embryonic development [52,53,54].

Sprouting angiogenesis is strictly regulated by the interplay between VEGF and Dll4/Notch signaling. This interplay is the basis for the lateral induction model, currently accepted as the prevailing mechanistic model explaining sprouting angiogenesis, and tip- and stalk cell selection. The supporting evidence for this was established in the post-natal retina developing vascular plexus. In response to spatial gradients of Vegfa, secreted by neuroglia cells migrating radially ahead of the vascular front, tip-cells sprout filopodia towards this gradient [7]. This effect is mediated by the interaction of Vegfa with Vegfr2 receptor, the concentration of which is especially high in tip-cells. Once tip-cells are selected and begin to move forward, formation of new capillaries begins because of the proliferation and migration of adjacent stalk ECs. When Vegfa gradients activate endothelial cells, they induce expression of Dll4 and Notch1 [55]. The tip-cell specific characteristics are preferably acquired by endothelial cells devoid of Notch1 and with high Dll4 expression. Dll4/Notch-associated transduction causes inhibition of sprouting by lowering ECs sensitivity to Vegfa. It was shown that in Dll4-hyperexpressing endothelial cells, expression of Vegfr2 was significantly inhibited [56]. Therefore, endothelial cells expressing Notch1 receptor, which was activated by adjacent Dll4 ligand, are prevented from transitioning to an active state, by lowering Vegfr2 levels, and thus Dll4/Notch signaling restricts the emergence of an excessive number of tip-cells, restricting excessive sprouting [57,58,59]. Specification of the tip/stalk cell phenotype by Notch is complex. In fact, even though Dll4 is the only ligand expressed in tip cells, Jagged1 and Dll1 are present in stalk cells [60]. Soluble Jagged1 was shown to reduce tip cell number, filopodia, and vessel density [59,61]. Moreover, Jagged1 was shown to have an opposite effect of Dll4 on branching morphogenesis, promoting endothelial cell proliferation and sprouting [62]. By using conditional *Jag1* loss- and gain-of-function mouse mutants, it was demonstrated that in contrast to Dll4, Jagged1 is proangiogenic and functions by downregulating Dll4–Notch signaling. In this study, it was shown that Jagged1 function is of particular importance in stalk cells, where Jagged1 levels are high and therefore efficiently antagonize the potent Dll4 ligand. Consequently, it confers upon stalk cells little ability to activate Notch in adjacent tip cells. Jagged1 was also demonstrated to counteract Dll4–Notch signaling interactions between stalk ECs, which helped to sustain elevated VEGF receptor expression at the angiogenic front. Therefore, in this region, ECs were still able to respond to VEGF, which, in turn, promoted proliferation and the emergence of new tip cells. These authors have additionally proposed that Fringe-mediated modification of Notch is of critical importance in regulating tip cell selection. These results were later confirmed in wound healing angiogenesis, also revealing that Jagged1 is expressed downstream of Dll4–Notch1 in stalk cells, creating a negative feedback loop that blocks tip-cell Notch1 from being activated [63]. The authors also found that stalk EC Jagged1 could activate both Notch4 in other stalk ECs as well as Notch3 in neighboring smooth muscle cells, positively driving differentiation of vSMCs and maturation of the nascent vascular network [63].

**Figure 2:**
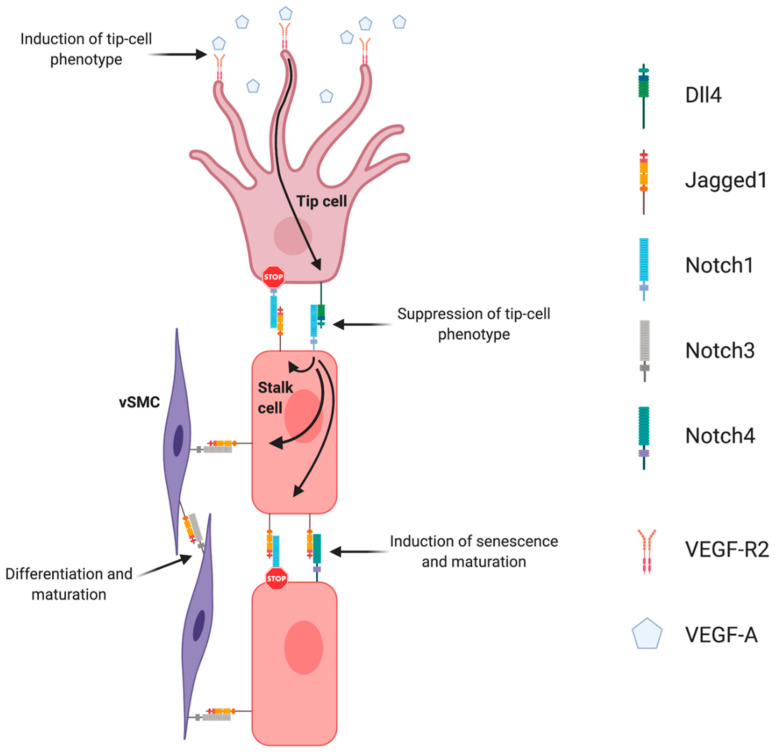
Notch signaling regulation of angiogenesis. Endothelial Dll4, mainly expressed in tip cells, activates Notch1 in adjacent stalk cells leading to the up-regulation of endothelial Jagged1. Jagged1 antagonizes Dll4 ability to bind to activate Notch1 in tip cells, creating a negative feedback loop in the regulation of endothelial branching. Endothelial Jagged1 positively regulates vascular maturation by two possible mechanisms: by activating endothelial Notch4 in stalk cells and Notch3 in vascular smooth muscle cells (vSMC).

The function that Notch signaling components play during developmental angiogenesis (Figure 2) was later confirmed to be mirrored in tumor neoangiogenesis. In 2006, two reports were published in Nature revealing the paradoxical phenotype of inhibiting Dll4 in restricting tumor growth [64,65]. In one report, the authors used soluble forms of Dll4, as well as antibodies to Dll4 that block the binding of mouse Dll4 to Notch1 receptor [64]. In the other report, a humanized phage antibody that binds with high affinity to Dll4, blocking the binding of mouse and human Dll4 to Notch1, was used [65]. Remarkably, the reduced tumor growth was associated with an increase in vessel density but an overall decreased perfusion. These two reports provided the first evidence that, for tumor development, the amount of vessels present is of less importance than the ability of the neo-vasculature to effectively deliver nutrients and oxygen to the actively dividing tumor cells [66]. This ability is provided in part by the maturation status of the newly formed vessels, and given that Dll4 loss-of-function mutants presented decreased mural cell recruitment, the vessel walls become leaky, and thus less perfused and functional [67,68]. By contrast with the VEGF blockade, Delta-like ligand 4 (Dll4) inhibitors resulted in increased tumor vessel density, characterized by sprouting and proliferating small vessel branches. However, these vessels were poorly functional, also resulting in decreased tumor perfusion overall and decreased tumor growth. These results introduced the concept that one could reduce tumor growth by “abnormalization” of tumor vasculature [66]. Although less effort has been put in studying other Delta-like ligands’ part in regulating the tumor vasculature, Dll1 has also been reported to be associated with tumor angiogenesis [69].

Jagged1 has also been studied in tumor development by its ability to regulate tumor angiogenesis. Genetic manipulation of endothelial Jagged1 in a mouse model of prostate cancer revealed that loss of endothelial *Jag1* had an inhibitory effect in the neo-angiogenic and maturation responses [70]. These results identified endothelial Jagged1 as a proangiogenic and pro-maturation regulator of tumor angiogenesis. Two other reports have suggested that Jagged1 expressed in tumor cells can stimulate angiogenesis. Firstly, Jagged1 expressed by cancer cells was dependent on activation of the mitogen-activated protein kinase (MAPK) signaling pathway. Thus, MAPK activation led to expression of Jagged1, which in turn influenced tumor neovascularization [71]. Secondly, Jagged1 expressed in breast tumor cells was shown to influence tumor angiogenesis [72]. 

The hypoxic environments, present in most cancers, may mediate Notch functions in tumor angiogenesis. A major mechanism by which cells respond to low oxygen tension is through the regulation of hypoxia inducible factor-1 alpha (HIF-1α). HIF-1α interacts with the Notch1 intracellular domain to augment responses to hypoxia downstream of Notch [73,74]. Dll4 expression by endothelial cells is also directly upregulated by hypoxia, possibly via HIF-1α and hypoxia response elements in the *Dll4* promoter [75]. 

Furthermore, a recent report using a Notch1 decoy, that specifically blocks both Jagged ligands mediated interactions, was shown to decrease xenograft growth by an anti-angiogenic effect and by the ability to destabilize pericyte-ECs interactions [76]. Moreover, the anti-angiogenic effect observed was likely due to increased secretion of the soluble form of Vegf-r1, and thus to decreased Vegf/Vegfr-2 signaling, suggesting that Jag1/Notch signaling is also able to positively regulate the VEGF pathway [76]. 

## 4. Notch Signaling Function in Solid Tumors 

Beyond the role of Notch signaling in tumor angiogenesis, it has also been implicated in the regulation of tumor cell proliferation and survival, in epithelial-to-mesenchymal transition, invasion and metastasis and in the regulation of cancer stem cells, in a variety of hematologic and solid malignancies [77]. Depending on expression patterns, the Notch pathway can be either oncogenic or tumor suppressive, even though the mechanisms are not fully understood [78]. These mechanisms may include tissue and cell dependent specific target genes, and varying cytokines and growth factors present in distinct microenvironments. Of these, breast (mammary gland) cancer, as a highly studied endocrine-dependent type of tumor, serves as a paradigm for understanding the effects of Notch signaling in solid tumors. One of the first clues that Notch signaling may play a role in solid tumors came from experiments with mouse mammary tumor viruses (MMTVs). Integration of the MMTV genome next to the ‘‘Int-3′’ locus resulted in a Notch4 activating mutation, which led to the constitutive activation of the receptor and breast cancer development [79]. Since this discovery, a number of studies have confirmed that activation of Notch signaling plays an oncogenic role in breast cancer. Inclusively, Notch1 and Notch3 oncogenic activity has been demonstrated in the mouse mammary gland [80] and, in humans, high levels of Jagged1 and Notch1 proteins have been associated with particularly aggressive breast cancer cases [81]. 

Abnormal regulation of the Notch pathway may occur by a variety of mechanisms including mutational activation or inactivation, overexpression, post-translational modifications, and epigenetic regulation [82]. For example, in ovary and breast cancers, somatic mutations, copy number alterations (The Cancer Genome Atlas Research Network), amplifications [83], and gene rearrangements [84] have implicated Notch pathway members in tumor pathophysiology. Moreover, Notch pathway regulation of tumor development can be closely related to other signaling cues involved in tumorigenesis, such as Akt, PTEN and NF-Kβ, as demonstrated in prostate cancer [85,86]. Akt lies at the center of a signaling node that phosphorylates a large number of substrate proteins and thereby regulates a number of potentially oncogenic activities, including cell survival via inhibition of Foxo transcription factors, activation of NF-kB, and phosphorylation of components of the caspase cascade.

During the transformation of a tumor in situ to an invasive carcinoma, epithelial tumor cells are released from their neighbors and breach the basement membrane barrier. The process underlying this phenomenon has often been suggested to involve EMT [87,88]. This is a unique process by which epithelial cells undergo remarkable morphologic changes characterized by a transition from epithelial cobblestone phenotype to elongated fibroblastic phenotype (mesenchymal phenotype) leading to increased motility and invasion [89,90]. Cells lose epithelial cell–cell junctions, cell polarity, actin cytoskeleton reorganization and the expression of proteins that promote cell–cell contact such as E-cadherin and γ-catenin, and gain the expression of mesenchymal markers such as vimentin, fibronectin, α-smooth muscle actin (SMA), fibrillar collagen (type I and III), fibroblast-specific protein-1, N-cadherin as well as increased activity of matrix metalloproteinases like MMP-2, MMP-3 and MMP-9 [91,92]. Notch signaling regulates the acquisition of the EMT phenotype [93,94]. Other key signaling pathways and molecules inducing EMT include Receptor Tyrosine Kinases (RTKs), the transforming growth factor beta (TGF-β) superfamily, Wnt and Hedgehog pathways [95] and NFκB [96]. Notch also cross-talks with several transcription and growth factors relevant to EMT, including Snail-1, Slug, and TGF-β. Snail-1 has been shown to be activated during EMT and act as a repressor of E-cadherin expression. The relation with Notch pathway was demonstrated by using immortalized endothelial cells that expressed Snail-1 under the overexpression of Notch-1, resulting in EMT and oncogenic transformation [97]. Notch signaling also controls Snail-1 expression under hypoxic conditions, both directly via transcriptional activation and indirectly via lysyl oxidase (LOX) [98]. In this context, EMT is promoted by direct regulation of *Twist* expression, through the induction of the hypoxia inducible factor 1-alpha (Hif1-α) [99]. Moreover, it has been reported that Jagged-mediated activation of Notch induces Slug expression, with subsequent repression of E-cadherin and EMT promotion [100,101]. Additionally, Jagged-1 or Hey-1 inhibition can arrest TGF-β- induced EMT [102]. 

Multivariate logistic analysis of renal cell carcinoma (RCC) specimens showed that tumor hematogenous metastasis not only depended on angiogenesis but also was associated with tumor size and DLL4 density. Functionally, the migration and invasion capacities of RCC cells were directly enhanced by DLL4–Notch binding. In addition, it was demonstrated that MMP secretion, involved in the basement membrane disruption and invasion, was Notch dependent because inhibition of the Notch effector Hey1 decreased both MMP2 and MMP9 [103]. In another study, Dll4–Notch signaling blockade was shown to inhibit liver micro-metastasis of human small cell lung cancer cells expressing high levels of *Dll4*. The authors suggest that was achieved by suppressing the early steps of liver metastasis through Notch1/NF-κB signaling attenuation, without significantly affecting angiogenesis [104]. MMGZ01, a novel human DLL4-specific monoclonal antibody, was found to inhibit tumor growth through various mechanisms, namely inhibition of tumor cell proliferation, promotion of tumor cell apoptosis and reduction in the cancer stem-like cell population, leading to the reversal of EMT [105].

Although Notch is not considered a strict “stemness” factor, in certain lineages Notch activation appears to favor maintenance or expansion of stem cell pools at the expense of differentiation, an activity with obvious potential cancer relevance. Notably, mouse models have consistently implicated Notch in maintenance of neural stem cells in the fetal brain [106]. These observations in developmental biology have led investigators to hypothesize that Notch might have a similar role in maintaining cancer stem cell populations, particularly in solid tumors such as glioblastoma [107,108], ovarian cancer [109,110], and breast cancer [111,112]. 

Multiple reports over the past decade have proposed that Notch has a role in promoting tumor metastasis [100,113,114,115,116,117] as well as resistance to both conventional chemotherapy [110,118,119] and targeted therapy [120,121]. 

## 5. Angiocrine Functions of Notch Signaling

**Figure 3:**
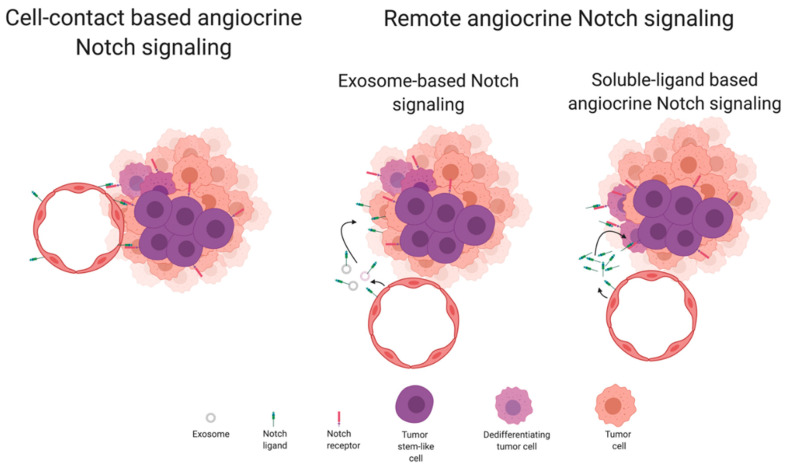
Modes of Notch-based angiocrine signaling in the tumor microenvironment. Cell-contact-based angiocrine Notch signaling occurs between ligand-bearing endothelial cells and Notch-receptor-bearing neighbouring tumor cells. Commonly, the end-result seems to be the induction of survival, stemness and invasion. Remote angiocrine Notch signaling occurs when endothelial cells release Notch-ligand-bearing exosomes or soluble forms of the extracellular region of Notch ligands. In the first case, the exosomal ligands can either activate Notch receptors in tumor cells or be incorporated in tumor cells before being functional. Doing so, they influence the tumor cell dedifferentiation process and tumor stem-like cells’ self-renewal.

Beyond the ability of Notch signaling components to regulate endothelial cell functions when present in the plasma membrane of endothelial cells, and the ability of Notch signaling components to regulate tumor cell functions when present in the plasma membrane of tumor cells, there are several reports of paracrine or juxtacrine activities for Notch components in the tumor milieu. These interactions can be of two types, by direct cell membrane contact, from tumor endothelial to neighboring tumor parenchima cells, or by indirect cell contact through the release of secreted soluble Notch ligands or Notch-bearing exosomes from tumor endothelial cells (Figure 3). As is common with Notch signaling, the cellular response varies with the tumor cell type and cellular context [122]. The range of responses can vary between alterations in survival or induction of chemoresistance, in invasive phenotype or induction of metastisation, or in induction of stemness. The regulation of these cellular responses is intertwined by signaling pathways’ interdependencies. This makes it difficult to assign a particular Notch pathway component to a specific tumor cell response. However, classical Notch signaling involves a stochastic event where cells with higher Notch ligand expression signal to neighboring stem-like cells to keep them from differentiating and continue proliferating, thus keeping their stem-like characteristics [106]. An example of this would be neurogenesis. In the case of cancer, the maintenance of stem-like characteristics and allowed proliferation of such cells has been, directly or indirectly, associated with the generation of metastasis. Since cancer stem cells are known for occupying a perivascular niche in the tumor, the study of angiocrine mechanisms of Notch signaling represents a strong proposition for the scientific community [123].

### 5.1. Remote Angiocrine Notch Signaling in Cancer

Remote angiocrine Notch signaling has been described through two different mechanisms. The first involves the secretion of a soluble truncated Notch ligand that diffuses in a gradient. These soluble Notch ligands have the ability to either activate Notch receptors in non-adjacent tumor cells or act as a dominant negative and block the ability of a given Notch receptor to undergo functional activation. There is no consensus on why, in some cases, a soluble Notch ligand acts as a functional ligand and, in others, acts as a dominant-negative ligand [124,125]. It is speculated that it could be linked with the base ligand itself or with the truncation point, enabling the soluble ligand to aggregate and form multimers [126]. 

In colorectal cancer, cancer stem-like cells are detected in the perivascular niche. Treating cultured primary CRC cells with EC conditioning medium expanded the cancer stem-like cells by increasing the dedifferentiation of cancer cells. It was also found that endothelial cell conditioning media pretreatment increased tumorigenicity and metastatic potential of xenografted primary colorectal cancer cell lines in vivo. It was found that this effect was mediated by Notch signaling and that the EC conditioning medium was differentially enriched for a truncated soluble form of Jagged1. Since knock down of Jagged1 or ADAM17, which is required for the truncation of Jagged1, eliminated the observed phenotype, the cause was attributed to the secretion of soluble Jagged1 from tumor endothelial cells [127].

The other mechanism involves Notch ligands being transferred from the cell membrane of a donor cell to the lipid membrane of an exosome. Again, it appears that Notch ligands present in exosomes can act by different ways. In some cases, the Notch ligands appear to be able to activate Notch receptors in cells along its diffusion path. In other cases, the Notch ligands appear to be transferred to receiving cells conducing to autocrine signaling.

Dll4 has been found in exosomes formed from tumor endothelial cells. In one case, exosomal Dll4 retained its activity as a functional Notch ligand, being still able to activate Notch receptors in signal-receiving tumor cells [128]. However, in a different study, the Dll4 bearing exosomes acted as dominant negative and blocked Notch signaling [129]. Interestingly, the same study found that both endothelial and tumor cells, that in that case also expressed Dll4, were capable of producing Dll4-bearing exosomes and that exosomal Dll4 was being incorporated into cells rather than signaling from exosomes to tumor or endothelial cells.

### 5.2. Cell-Contact-Based Angiocrine Notch Signaling in Cancer

Cell-contact-based Notch signaling represents the classical view of Notch signaling. This means that both the ligand and receptor are present in adjacent cell membranes and the signal receiving cell makes a cellular decision. This decision is resultant from changes in its transcriptional pattern secondary to the translocation of the Notch intracellular domain from the plasma membrane to the nucleus. Endothelial cells communicate through Notch pathway components to tumor cells that reside in the so-called vascular niches, that are easily formed in tumors by the tortuosity of its vasculature [123]. These vascular niches comprise clusters of tumor cells that are tightly surrounded by tumor endothelial cells, and due to the close proximity, can be involved in cell-to-cell signaling. The vascular niches frequently contain, or are enriched for, cancer stem-like cells. In the mouse prostate, endothelial Jagged1 was found to activate Notch3 in neighboring tumor cells, which has been associated with poor prognosis and high metastatic potential. The result of such activation was found to be increased cellular proliferation, upregulation of *Tgfb1* and induction of EMT [70]. In another study, tumor cells were found to induce an incomplete endothelial–mesenchymal transformation (EndMT), contributing to the generation of a pro-tumoral niche [130]. Again, these tumor endothelial cells, in return, promoted tumor growth and stemness in the neighboring tumor cells. Analysis of the tumor endothelial cells revealed that Jagged1 and TGF-β are likely the main contributors to this effect [130]. In lymphoma, Jagged1 was found to be upregulated in tumor endothelial cells in response to secretion of FGF4 by B cell lymphoma cells. Upregulation of *Jagged1* in tumor ECs leads to increased activation of Notch2 in lymphoma cells, which take on a more aggressive phenotype, that is more stem-like, invasive and displays increased chemoresistance [131]. In breast cancer, endothelial Jagged1 was found to activate Notch1 in neighboring breast cancer stem cells, upregulating *Zeb1*, which in turn increases Vegfa production and further activates the tumor neovasculature. This positive feedback loop was found to promote tumor growth [132]. In glioblastoma, glioma stem-like cells are detected predominantly in the perivascular region. Using a stem/progenitor cell-fate tracking reporter system, it was detected that differentiated tumor cells dedifferentiate to glioma stem-like cells only in perivascular regions and display increased levels of NICD and Notch downstream effector ID4 [133]. In addition, using a co-culture system of human brain microvascular endothelial cells with glioblastoma multiforme neurospheres increased cancer stem-like cells’ self-renewal dependent on endothelial Dll4 and Jagged1 function [108]. Applying the same principle in vivo revealed that intracranial xenografts grew smaller when the initial inoculum included endothelial cells depleted for Dll4 or Jagged1, due to a concomitant reduction in cancer stem-like cells [108]. In colon cancer transplant mouse models, Dll4 and Jagged1 presence in tumor endothelial cells was found to activate Notch1 in neighboring tumor cells. Notch1 activation led to increased ability to perform trans-endothelial migration and intravasate, thus promoting metastasis [115]. Notch3 expression is known to be amplified in ovarian cancer and associated with its progression. Activation of Notch3 has been associated with ovarian cancer cells adhesion to peritoneal cells and cancer cell metastatic outgrowth. It was found that this effect was dependent on the presence of Notch ligands in the tumor vasculature, namely Jagged1 [134]. Another study demonstrated that targeting stromal cell-derived, but not tumor-derived, Dll4 reduces ovarian xenograft tumor volume. Using RBPj-Notch reporter activation as an indicator for Notch activation and combining with specific Notch receptor antagonists, it was found that this effect proceeded from endothelial Dll4 signaling through Notch1, but not Notch3, in neighboring tumor cells [135]. In T acute lymphoblastic leukemia (T-ALL), an aggressive neoplasm of immature T-cells, Notch3 is commonly overexpressed but is not constitutively activated as Notch1 normally is. Dll4 was found to be expressed in ECs of growing but not in dormant tumors. Concomitantly, Notch3 was also found to be overexpressed in more aggressive tumors. Endothelial Dll4 was found to contribute for Notch3 activation in T-ALL cells, delivering a survival signal [136]. Growing xenografted tumors in endothelial–specific *Dll4* loss-of-function mice revealed that despite being highly hypoxic, these tumors displayed reduced numbers of cancer stem cells and reduced levels of EMT, when compared to mice with normal expression of *Dll4*. In turn, this led to reduced numbers of circulating tumor cells and lung metastases [137]. Another way through which the endothelial Notch pathway activation can influence the progress of a tumor cell is by inducing the secretion of soluble factors or expression of membrane proteins that act paracrine. VCAM1 was identified as a Notch1ICD direct target in tumor endothelial cells that positively regulated the opening of EC junctions and homing for tumor cells, facilitating transmigration and colonization [138].

## 6. Conclusions

Although much is still unknown, it is becoming clearer that the tumor vasculature cannot continue to be seen as a passive conduit for blood perfusion, answering only to the tumor needs. Instead, the tumor vasculature is an active tissue that answers back to the tumor and positively supports tumor growth, independently of its primary function. In a way, despite not being transformed, the tumor endothelial cell acts to support the growth of its own tissue in a way that is not too dissimilar to what a tumor cell does. It makes use of developmental gene pathways to positively influence neighboring cells, which will in turn positively influence its own growth. The Notch signaling pathway seems to be key here. Due to the way it can influence cellular response to growth factors, it has the ability to perform cellular conditioning, controlling the cellular decision-making process in its own interest. Although Notch signaling response is very context-dependent, this also means that anti-angiogenic therapies, that have largely been studied as acting on a passive tissue, should be reevaluated in light of this knowledge. An initial concern for antiangiogenic therapies’ side-effects was that they could lead to increased invasion and metastasis by increasing hypoxia-driven EMT. Now we know that, for instance, removing Dll4 from tumoral endothelial cells has the ability to block this exact effect [137]. It is time for increased study of the autocrine and angiocrine functions of tumoral endothelial cells and to turn gained knowledge into novel therapies to more effectively target Notch signaling in cancer treatment.
